# Robot System Assistant (RoSA): Towards Intuitive Multi-Modal and Multi-Device Human-Robot Interaction

**DOI:** 10.3390/s22030923

**Published:** 2022-01-25

**Authors:** Dominykas Strazdas, Jan Hintz, Aly Khalifa, Ahmed A. Abdelrahman, Thorsten Hempel, Ayoub Al-Hamadi

**Affiliations:** Neuro-Information Technology, Otto-von-Guericke-University Magdeburg, 39106 Magdeburg, Germany; jan.hintz@ovgu.de (J.H.); aly.khalifa@ovgu.de (A.K.); ahmed.abdelrahman@ovgu.de (A.A.A.); thorsten.hempel@ovgu.de (T.H.)

**Keywords:** augmented reality, activity recognition, cooperative systems, facial recognition, gesture recognition, human-robot interaction, interactive systems, robot control, speech recognition

## Abstract

This paper presents an implementation of RoSA, a Robot System Assistant, for safe and intuitive human-machine interaction. The interaction modalities were chosen and previously reviewed using a Wizard of Oz study emphasizing a strong propensity for speech and pointing gestures. Based on these findings, we design and implement a new multi-modal system for contactless human-machine interaction based on speech, facial, and gesture recognition. We evaluate our proposed system in an extensive study with multiple subjects to examine the user experience and interaction efficiency. It reports that our method achieves similar usability scores compared to the entirely human remote-controlled robot interaction in our Wizard of Oz study. Furthermore, our framework’s implementation is based on the Robot Operating System (ROS), allowing modularity and extendability for our multi-device and multi-user method.

## 1. Introduction

Recently, collaborative robotics (cobots) has experienced increasing popularity as it is targeted to be a more flexible and general task type of robot [1]. Compared to conventional industrial robots, cobots share their work area with humans and interact directly. This requires new standards of safety and interaction interfaces. Typically, robots are instructed by buttons, knobs, joysticks, specific speech, and gestures commands, teaching through touching and guiding or dedicated teaching panels. Either way, the handling of the robot requires prior knowledge and is not intuitive for untaught users. Therefore, the interaction interface must shift to a more human-centered and adaptive relation to enable the use of cobots in an unconstrained environment with varying tasks and interchanging human collaboration partners.

There have been multiple promising research approaches tackling flexible Human-Robot Interaction (HRI) scenarios [2,3,4,5], but most of their methods are driven by the introductions of new techniques instead of focusing on the needs and characteristics of interaction patterns from a human perspective.

With the aim of a better understanding of human behavior in robotic interaction scenarios, we carried out an extensive Wizard of Oz (WoZ) study [6] to examine common communication intuitions of untaught human interaction partners. In addition, we worked out human key actions to approach human-robot interactions. On this basis, we conceptualized and implemented a new multi-modal robotic system called “RoSA” (Robot System Assistant) that tackles the challenge of intuitive and user-centered human-robot interaction by facilitating multiple input streams such as speech, gesture, face, body, and object recognition. The use of a wide range of perception capabilities combined with speech processing ensures a robust scene and interaction state estimation and leads to an efficient and intuitive human-robot collaborative task performance.

In this paper, we tackle the challenge of turning the results from our prior WoZ study into a fully autonomous robotic systems that can handle interaction with untaught human partners without external control. First, we derive a concept that enables the robot visual and acoustic perception of potential human interaction partners and the scene understanding. We show which features are crucial for perceiving interpretable indications to derive meaningful instructions for the robot. We continue with an in-depth analysis of each module and its interplay with the core system and present further implementation details. Finally, the evaluation of our RoSA system is conducted via a separate study similar to our previous Wizard of Oz study, but this time with a fully autonomous robotic system.

## 2. Related Work

**Figure 1:**
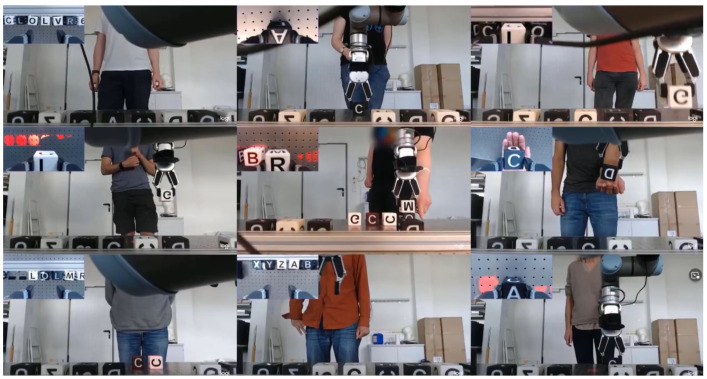
Previous field study for natural human-robot interaction using the Wizard of Oz method. A video summary can be found here: https://youtu.be/JL409R7YQa0, (accessed on 18 January 2022).

In our earlier Wizard of Oz (WoZ) study [6], we reviewed different interaction modalities required for an intuitive HRI. The participants were permitted to use different features like gestures, speech, mimics, and gaze without any limitations to communicate with a cobot and execute different tasks like cube stacking. We tricked the subjects into thinking they were interacting with a state-of-the-art artificial intelligence controlling a cobot, whereas in fact, we were remote controlling the cobot based on the participant’s instructions and a strict set of rules. This principle is commonly called a Wizard of Oz experiment. Figure 1 shows multiple views of the subjects interacting and giving orders through speech or gestures.

It was shown that 97% of the 36 subjects used speech and 75% used pointing gestures to solve the given tasks. Most of the subjects preferred the path planning to be done by the robot assistance system and did not want to guide the robot directly but give more complex commands.

In regard to safety, by complying with the standards, ISO 10218-1/2 [7] and ISO/TS 15066 [8] on HRI, the danger to the user is minimized. As Pasinetti et al. [9] have shown, time-of-flight (ToF) cameras can be used to detect the operator and, in combination with virtual barriers, slow or stop the robot when safety guidelines are infracted. In addition, one of our previous implementation of a gadget-less HRI concept Robo-HUD suggests that a non-contact approach also contributes to a safe HRI [10]. An attention module based on head posture estimation was introduced to monitor attention, allowing intended user actions.

It also introduced an *Attention Module* based on head pose estimation to monitor awareness, enabling the interaction only when intended. This module allows users to switch between workstations without logging in or out when used in a multi-device scenario.

Many implementations already exist in this area  [11,12,13,14,15,16,17,18,19]. Magrini et al. [20] proposed a system that ensures human safety in a robotic cell. It is based on the method of Pasinetti et al., which uses ToF cameras to localize users in real time. In addition, their system enables gesture recognition for low-level robot control (e.g., start/stop). These gestures are two-handed gestures that must be clearly shown above the head. This type of gesture is already built into the Kinect V2 software and is known to be relatively robust. Additionally, facial recognition can be used to personalize and create a long-term user experience [21].

Based on these findings and due to the lack of a state-of-the-art implementation of an intuitive multi-modal overall concept, the Robot System Assistant (RoSA) concept was created [22].

## 3. Framework Overview

### 3.1. Concept

**Figure 2:**
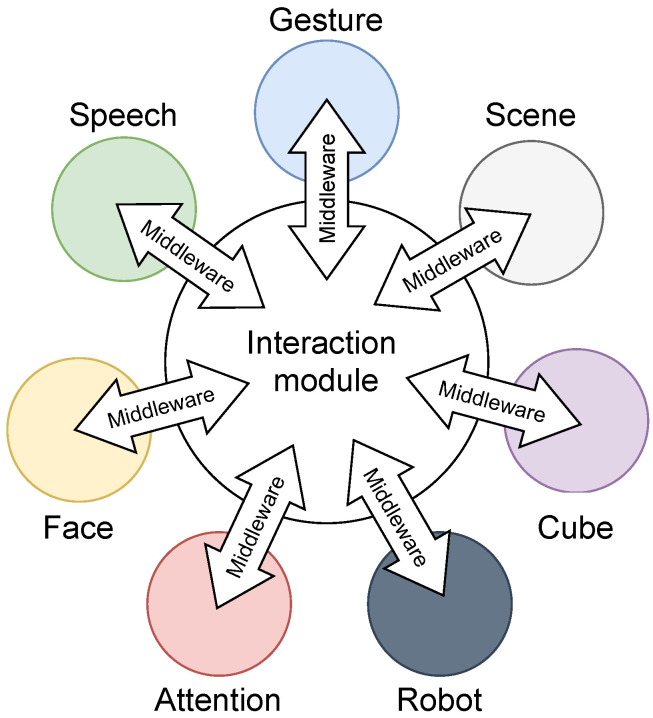
Concept: The interaction module connects through middleware to other modules.

We worked out a concept to assemble all important information streams into synergetic interplay. The overview can be seen in Figure 2. It consists of seven modules (face, attention, speech, gesture, robot, scene, and cube) communicating through middleware to a core unit, the interaction module, which is responsible for the logic and actions of the system. The modular approach allows an independent development and evaluation of each necessary component. While the functionality for most of the modules is self-explanatory, the *Scene Module* and *Cube Module* require a more detailed introduction. The *Scene Module* is responsible for the data storage from the virtual objects and contains the constraints, positions, and calibration of the system. In this module, a digital twin of the scene gets depicted.

The idea of using cubes as an interaction object was carried over from the experimental design of preceding studies in order to stay consistent and to allow a direct comparison with the WoZ study. The cube module takes care of the cube logic and their detection.

For scalability purposes and to evaluate a realistic scenario, the modules designed to be able to run as multiple instances, allowing a multi-device setup. The individual devices, the middleware, and functions of the mentioned modules are described in detail in Section 4 and Section 5.

### 3.2. Features

In the previous studies RoSA was a system that was capable of speech, gesture, face, body, and attention recognition based on the “wizard”, the operator controlling the robot. To automate the system and combine the cobot with artificial intelligence, a set of necessary features was elaborated, which is shown in Table 1.

**Figure 3:**
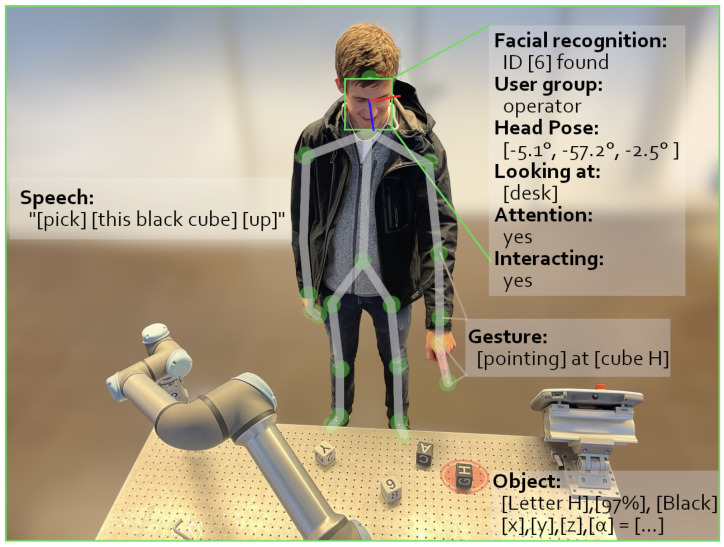
User pointing at a letter Cube. Multiple detected features are displayed for clarification.

The detected features are depicted in Figure 3 showing an exemplary situation of a user interacting with the system.

## 4. System Setup

### 4.1. Hardware

We chose a modular structure in the form of so-called workstations (WS) to be capable of evaluating the system in a multi-device scenario and to simplify the development and the integration of additional hardware. A WS is mainly characterized by its hardware, which forms a closed system.

For consistency and comparison purposes, we did not change the hardware used in the previous studies. As cobot, the UR5e industrial robot equipped with an *RG6* gripper was used. The robot was bolted to a sturdy metal table. A TV, with a ToF (Kinect) camera on top, was placed behind. A projector, lighting the scene from above, was used to illuminate the objects and the metal table for visual feedback. The same cubes, as in the previous WoZ study, were used. We refer to this cube-related setup as workstation 1 (WS1).

**Figure 4:**
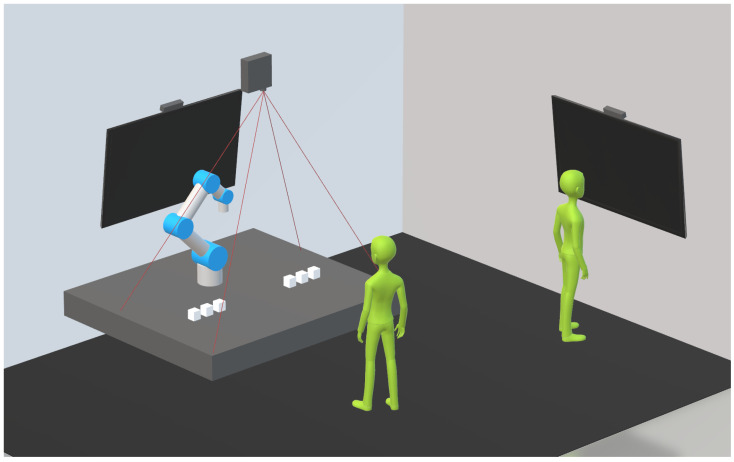
A schematic overview of the workstations.

The second workstation (WS2) can be used for registration and questionnaires, featuring a smart screen with a touch function and also a ToF (Kinect) camera. Every workstation utilizes microphones (from Kinect) and speakers (from TVs). The current setup can be seen in Figure 4.

### 4.2. Middleware

The development of a distributed system consisting of heterogeneous devices from different manufacturers is a non-trivial task that, among other things, must ensure communication between numerous devices. The Robot Operating System (ROS) is used for communication between the hardware components as well as the individual software modules. For communication within the *Speech Module*, the Message Queuing Telemetry Transport (MQTT) protocol was implemented. Using ROS as Middleware grants a direct machine-to-machine communication interface and allows an easy integration of additional workstations. Each module can be run as an independent node multiple times on multiple devices.

For communication between modules, a set of custom ROS messages were made: *Body*, *Joint*, *Face*, *RobotAction*, *CubeAction*, and *CubeMessage*. The code is open source [31].

## 5. Modules

### 5.1. Scene Module

This module creates a virtual scene that contains all virtual objects and their relations, enabling the interaction and management of the objects. The scene can be an exact or an abstract representation of the real environment, or it can create an entirely new one.

The *Scene Module* also includes the calibration of the system’s input and output devices. Recognition algorithms can then be used to associate real objects in the virtual world. In the context of HRI, the *Scene Module* is also used for collision calculations and as a database for object positions.

The table on which the experimental setup of WS1 is located serves as the basis for the scene and calibration. This table is provided with threads at regular intervals of 2.5 cm, which form a grid. This grid allows for calibration between the robot and scene, as well as proper alignment of the projector. In this case, the calibration is an adjustment and scaling of the respective coordinate systems in relation to each other. The point where the projection of the pixel [0,0] meets the table surface serves as the origin of the coordinate system in the virtual world. This is scaled according to the grid.

All virtual objects are defined by a point that marks their position in the virtual world in Cartesian coordinates.

#### 5.1.1. Skeleton

In addition to the cubes, the data of the Kinect skeleton is also converted into virtual objects, consisting of 26 [x,y,z] points. This allows the user to interact with virtual objects such as security planes or augmented user interfaces.

#### 5.1.2. Spot

**Figure 5:**
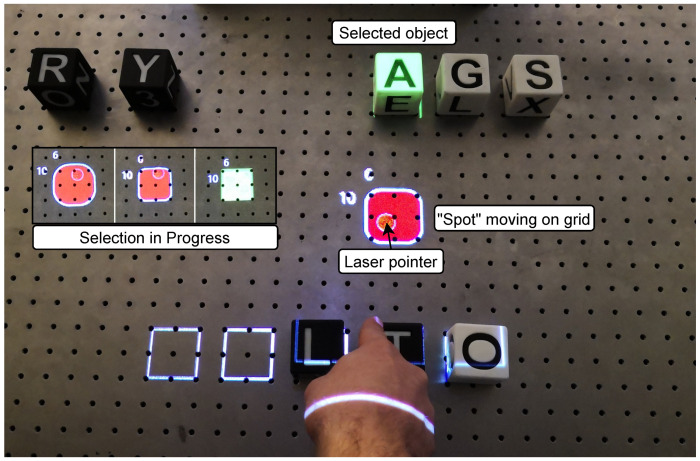
Selection in progress: The borders of the grid rectangle around the laser pointer are narrowing. In the next step, the selection would be moved from *Cube A* to the new coordinates.

The pointing gesture creates a so-called *Spot* at the point where the line, through the elbow and wrist of the Kinect skeleton, intersects the surface of the grid. It contains the position of the intersection point [x,y,0]. To help the stacking of the cubes, the spot jumps to the nearest grid position. The *Spot* can be seen in Figure 5.

#### 5.1.3. Visual Feedback

Through monitors or projection, virtual objects can appear in reality. A selected virtual cube is illuminated with a green rectangle, and a space on the grid is illuminated with a white rectangular frame. Transformations are applied to the projection so that the projected objects visually match the real objects. The exact position of the *Spot* is projected onto the table like a laser pointer. The process of selecting an object and the projected laser pointer can also be seen in Figure 5.

#### 5.1.4. User

The user database includes the ID, names, facial features, and session status of the subjects. WS1 and WS2 can both access this database to address the user by name, or to retrieve the last session status for the activated subject. This personalized experience contributes to the intuitiveness of the system.

### 5.2. Robot Module

The *Robot Module* is responsible for path planning and managing robot actions. Path planning is an important feature of a robot assistant. For this task, the definition of start and end points, from now called *Source* and *Destination*, is crucial.

**Figure 6:**
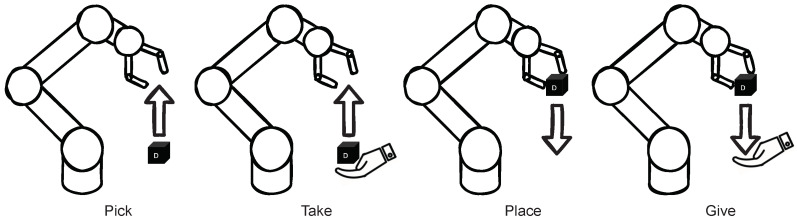
Basic robot operations: Pick up, take from user, place on table, and give to user.

The *Robot Module* sends commands, so called *RobotActions* to the robot. The robot can move to the discrete position in the virtual grid and grasp real objects. For this purpose, there are four basic operations within the robot program (see Figure 6). These can be defined as individual modes for programming the cobot. These sub-routines can be called directly via Real-Time Data Exchange (RTDE)—a protocol developed by Universal Robots for fast communication with the robot. A thread observes a particular register and jumps to the corresponding mode called by the *Robot Module*. When performing the *pick* or *place* operation, the appropriate mode must be specified, as well as the desired coordinates of the object. If a cube is to be *taken* or *given*, only the mode and position of the hand are necessary. The four basic operations, require only either a *Source* or *Destination* (i.e., object or hand position), since the respective counterpart, logically results itself as an actuation of the gripper. These operations execute only a single step. The more complex commands like *picking and placing* are further explained in Section 5.3.3. In addition to the four basic modes of interaction with the cubes, there are five more modes that complete the robot program.

- Abort: Motion is aborted;
- Home: Robot goes to initial position;
- Sleep: Robot goes to idling position;
- Toggle gripper: Opens or closes the gripper;
- Greet: Robot performs nodding motion.

To ensure that all commands are registered correctly, the robot changes its state to *busy* during an action. When a robot action is finished, the robot changes its mode to *Ready* and continues to execute the *RobotActions* that have queued up.

### 5.3. Cubes Module

#### 5.3.1. Physical Cube

The interaction at WS1 is primarily with 5×5×5 [cm] cubes, that can be positioned exactly on the 2.5×2.5 [cm] grid of the table. The cubes have a letter on each side and are 3D-printed from lightweight and robust Polylactic Acid (PLA), allowing for safe interaction. The cube model can be downloaded from Thingiverse [32].

Each real cube is represented by a virtual object, which is given the attributes: Letter, color, and position. The cubes are unique and thus can be identified by one or the combination of the attributes. In summary, the cube data is stored as an ROS message of type:

CubeMessage { letter [A-Z], color [black/white], position [x,y,z] }.

When a cube is handed over, it is assigned the position [0,0,99] until it is reassigned a coordinate on the table by an interaction with the robot. If a cube is in the gripper, it is assigned the coordinates [0,0,-1]. All other possibilities [x,y,z] correspond to a position on the grid, where x, y, and z take real values.

#### 5.3.2. Cube Detection

For an unconstrained interaction, the robot must be continuously aware of the position and order of the cubes placed on the desk. A straightforward approach would be the use a visual tracking method to follow the cube operations and update its current position accordingly. However, occlusions caused by the robot and cubes that are leaving the field of vision can heavily harm the tracking state and lead to interaction discontinuities. We therefore follow the approach of continuous detection and recognition of each cube and adapt a deep neural network for this purpose.

**Figure 7:**
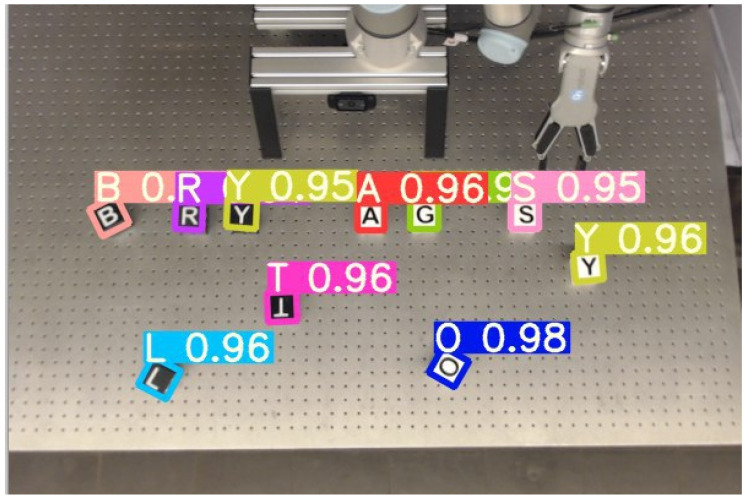
The cube detection in action. It detects also the rotated orientation of the cubes.

Figure 7 shows an example image of our cube detector in action. It demonstrates that even the almost 180° degree rotation of the cube “T” can be detected precisely with a high confidence (shown as a number next to the letter).

As there is no public dataset available that would fit our needs for the cube detection task, so we generated our own synthetic dataset. We cropped representative cube images for each letter and color and randomly placed them on arbitrary image backgrounds. Additionally, random rotations and scaling as well as different levels of blurring are applied to further augment the variance of the data. As a result, we received 100,000 randomly generated training images with corresponding annotation. Beside the ground truth letter and localization, we also annotated the rotation of the cubes as this information is crucial for accurate grasping by the robot.

For implementation, we use the Yolov5 networks as a backbone and change the number of output neurons to the number of letters on the cubes. Instead of regressing the cubes rotation directly, we found that classifying the angle leads to more stable results. Therefore, 90 classes are added for each degree of rotation. We used rotated bounding boxes for calculating the Intersection over Union to further help the network to learn the rotation. The cube detection network is open source [30].

#### 5.3.3. Cube Logic

**Figure 8:**
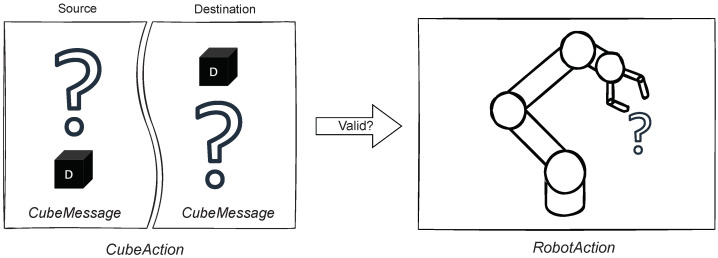
Conversion of *CubeAction* to *RobotAction*.

The Cube Module is an indispensable additional module that is used to check the interactions with the cubes, convert them into an *RobotAction*, and change the virtual cubes in the scene. Figure 8 shows the build of *CubeAction*, a ROS Message type consisting of two *CubeMessages* describing a source and destination. The task of the *Cube Module* is to find the transition from source to destination and convert it to a *RobotAction*, if the transition is valid and reasonable according to the *Scene Module*.

Every *CubeAction* has a source and destination. If either of the *CubeMessages* equals the robot gripper, then this *CubeAction* corresponds to a basic *RobotAction* discussed previously in Section 5.2. It is a single step of the robot moving the cube to or from the gripper. In the previous WoZ study, the users requested the robot to move the cubes between positions, disregarding the intermediate steps. Thus, the necessity of complex or combined instructions became eminent.

**Figure 9:**
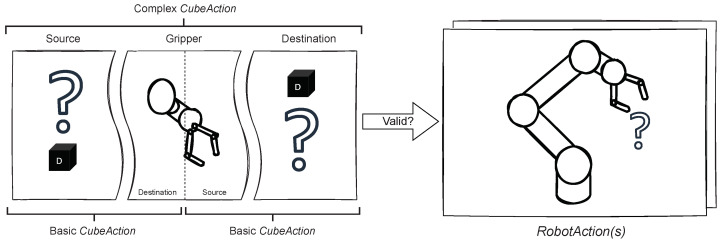
*CubeAction* can be simple or complex, corresponding to multiple *RobotActions*.

Two basic *CubeActions* can be chained together, if they have the same intermediate position, i.e., the gripper. Figure 9 depicts this concept.

**Figure 10:**
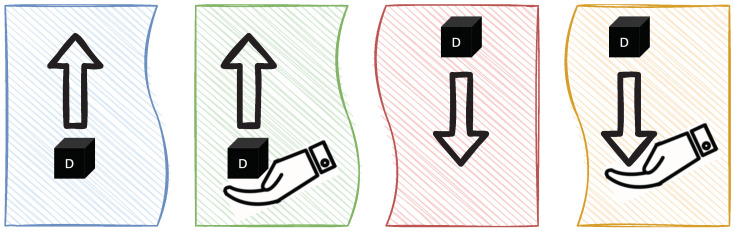
Basic operations Source: {*Pick*, *Take*} and Destination: {*Place*, *Give*}.

For the *Interaction Module* and *Speech Module* to function correctly and as expected, it is necessary to consider, identify, and group all possible cube manipulations and their corresponding *RobotActions*. Thanks to the complex *CubeAction* concept, it is possible to list all possible combinations. Figure 10 shows the four basic robot operations (see Figure 6) *Pick*, *Take*, *Place*, and *Give* depicted as either source or destination *CubeMessage*.

**Figure 11:**
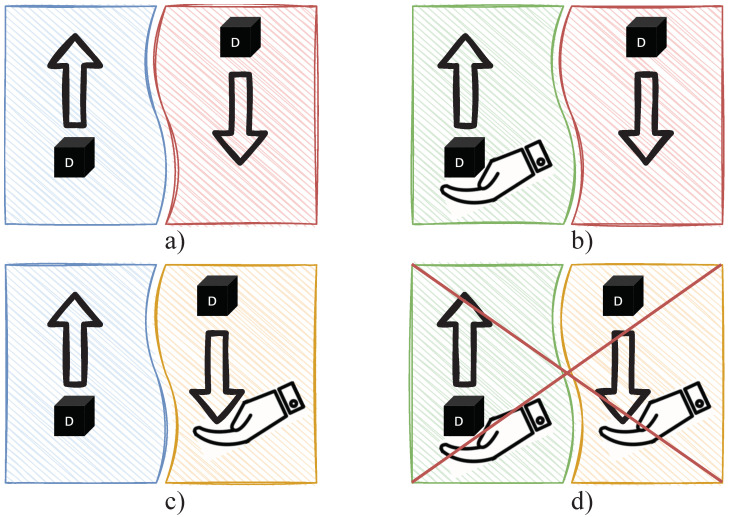
Complex operations: (**a**) Pick-Place, (**b**) Take-Place, (**c**) Pick-Give, and (**d**) Take-Give.

These operations can be combined to form four complex operations, as depicted in Figure 11. *Pick-Place* is used to move a cube from one position to another. *Take-Place* is used to receive the cube from the user and place it on the table. *Pick-Give* is used to give a cube after picking it up. *Take-Give* action is logically exclusive, since the object does not change its position from the system’s perspective and thus can be neglected.

The source and destination cube may be specified by any combination of the attributes position, color, and letter. The *Cube Module* then checks the start and target position by matching them with the cubes stored in the scene. If no position but color and/or letter was specified as a start cube, then the corresponding position is retrieved from the scene and the information is added to the *CubeMessage*. Contradictory *CubeMessages* are filtered out. When a *CubeAction* is verified, it is converted into one or, in the case of complex operations, two *RobotActions* and sent to the *Robot Module*.

The four basic operations always include the gripper (position [0,0,-1]) as a source or destination. An example of a *CubeAction* for *Place*, that puts the cube that is currently in the gripper to position [12,12,1], could look like this:
Src.{letter[],color[],position[0,0,-1]}Dst.{letter[],color[],position[12,12,1]}.

This message would be then checked by the *Scene Module* and then converted to a *Robot Action* and enqueued by the *Robot Module*. After the successful movement of the robot, the *Robot Module* would report the new position, and the corresponding cube in the *Scene Module* would be updated:

CubeMessage { letter "M", color "white", position [12,12,1] }.

All other cube manipulations are executed in the same manner. The remaining challenge for the *Interaction Module* is to combine the correct information from the *Speech Module* and *Gesture Module*. A complex *Cube Action Pick-Place*, for picking a black cube “A” and placing it on top of cube “B” could look like this:
Src.{letter"A",color"black",position[4,8,1]}Dst.{letter"B",color[],position[6,10,2]}.

In addition to checking whether a field is already occupied, the fields in the immediate vicinity are also checked. If these are also occupied, it is possible to place a cube on top between two cubes, thus stacking a pyramid. If no neighboring cubes are present, a cube can only be placed directly on another one. Vice versa, whether there is another cube above the selected one is also checked.

The module is also able to move the cubes back to their initial position. For this purpose, corresponding *Cube Actions* are created based on the scene. The order of the stacked blocks is also taken into account.

### 5.4. Face Module

The human identification serves both security and personalization of the data presented. Moreover, face verification is a vital identity authentication technology used in more and more mobile and embedded applications. Our system benefits from face verification to achieve high fidelity and confidence for user authentication and authorization to control the robot for crucial tasks.

The *Face Module* is subdivided into two main parts: Face detection and face recognition. The face detection can detect the location of the face in any input stream (image or video frames). The output is the bounding box coordinates and facial landmarks of the detected faces. On the other hand, face recognition is a process that compares multiple faces to identify which face belongs to the same person. This identification process can be done by comparing the feature vector of the detected face with the stored face feature vectors.

#### 5.4.1. Detection Part

We used the RetinaFace [25] light-weight model, based on MobileNet-0.25 [33], as the pre-trained model for face detection, which employs a multi-task learning strategy to simultaneously predict face score, face box, and five facial landmarks. The network was pre-trained on a WIDER FACE dataset [34].

#### 5.4.2. Recognition Part

After the detection bounding boxes are obtained, the filtered boxes are fed into the recognition part. Before the next steps, the Practical Facial Landmark Detector (PFLD) [35] is used to align the detected faces. The aligned faces are used for both face recognition and facial expression recognition.

**Figure 12:**
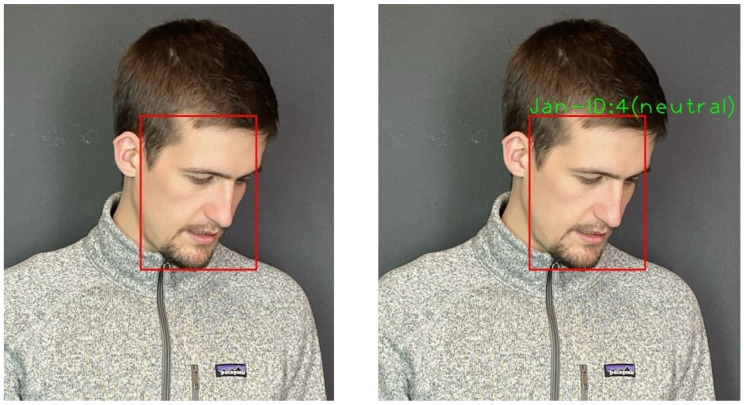
Face detection, face recognition, and facial expression recognition.

For face recognition, the deep CNN used was MobileFaceNet [36] which uses less than 1 million parameters and is specifically tailored for high-accuracy real-time face verification on mobile and embedded devices. This is less accurate than its counterparts, but it is real-time capable. The network was trained on the refined MS-Celeb-1M dataset using the loss function Arcface [23]. This loss function produces much better discriminative features compared to others. The extracted facial features are compared against each other using cosine similarity. If the faces are similar enough, they are assigned the same ID (see Figure 12). The IDs in the image section are passed to the *Interaction Module* along with their position. Persons who cannot be assigned an ID from the database are assigned the ID −1.

### 5.5. Attention Module

Various features can be used to estimate the user’s intention to interact with the robot (attention) and the intention breakdown during the interaction (attention breakdown). The most common features that can be used to estimate engagement and disengagement in HRI include gaze, head pose, face, posture, speech, and distance [37,38,39,40,41]. Using more features to estimate user attention could increase the accuracy of the attention algorithm. However, it will increase the computational cost, which will have a bad impact on the overall system.

The direction of a person’s gaze has a regulative function for the interaction taking place and allows conclusions to be drawn about the willingness to interact [42]. The direction of gaze can be recognized by the position of the head but also by the pupils. The latter gives more precise information about the current visual focus. If no fast or small gaze changes are required (e.g., when reading) and the object viewed is in the middle range of the visual angle or the person is further away, the orientation of the head offers a possibility for approximation [43].

Considering attention in the context of a technical system, a POI can be defined. If a person turns away too far from the POI, the person likely has no longer potential engagement. If the camera is in the POI and the user’s head is in the center of the frame, a deviation in the yaw-pitch-roll angle results from the subject turning away, thus breaking the engagement. However, when the relative position in the image plane is changed, a transformation must take place to determine whether that person is looking at the POI.

**Figure 13:**
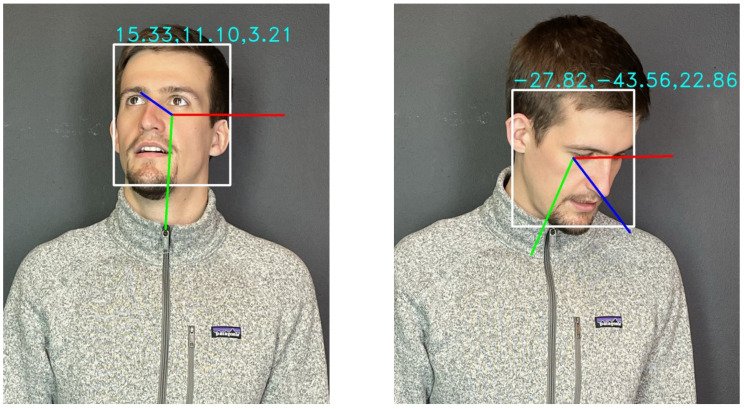
Head pose detection.

In our algorithm, we fused the head posse features with the gaze features through a rule-based classifier to estimate the person’s attention while interacting with the robot. For the head pose features, we used the img2pose method proposed by Albiero et al. [44]. This method outperforms many current state-of-the-art models in terms of accuracy and real-time capability. The method does not use the elaborate detected bounding boxes and landmarks, but uses a Faster-R-CNN-based model that computes the 6-Degrees of Freedom pose for all faces in the photo (see Figure 13). The model used, was trained using the WIDER FACE dataset. For the gaze features, we used the gaze360 method proposed by Kellnhofer et al. [45]. They uniquely take a multi-frame input (to help resolve single frame ambiguities) and employs a pinball regression loss for error quantile regression to provide an estimate of gaze uncertainty. This method is trained on 3D gaze in-the-wild dataset, which make it robust to diverse physically unconstrained scenes.

An algorithm converts the resulting head pose and gaze features into a person-based visual attention score for each person in the scene. We fused these scores together through our algorithm for outputting a final score for each person in the scene. If the predefined threshold of visual attention focus is exceeded, the person is recognized as attentive and can interact with the robot. If the person turns away, the visual attention focus decreases over time. If a threshold value is undershot, this person is no longer detected as attentive.

### 5.6. Gesture Module

The pointing gesture uses the skeletal data of the forearm provided by the Kinect in combination with the “index finger pointing” gesture (Kinect Lasso gesture). According to the concept of “Laws of Linear HRI” [12], a line is formed from the two joints, elbow, and wrist, of the recognized human and the intersection with the plane of the table is determined, the aforementioned *spot*.

The lack of direct user feedback can be solved by adding a laser pointer feature that directly corresponds to the pointing position. This way, the user does not have to wait for the robot to fulfill the task, as in the case of the original implementation by Williams et al. [46]. Using the *spot*, the user is currently pointing at, as real-time feedback, further helps to increase the overall accuracy of the pointing gesture.

**Figure 14:**
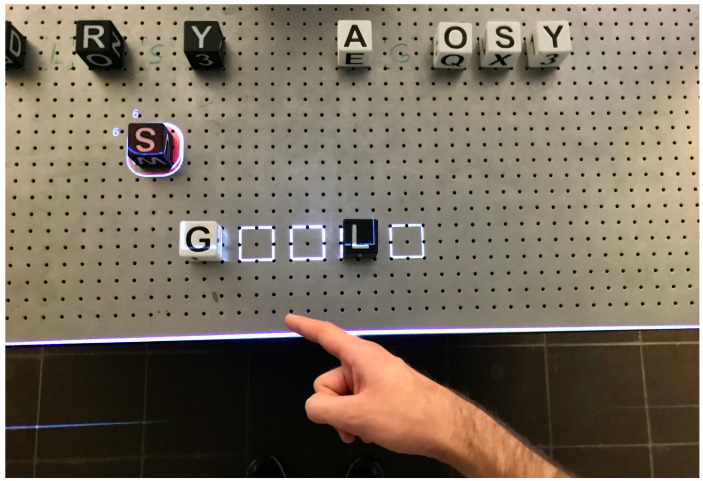
Pointing gesture: User points to cube [S], which is enveloped by the laser pointer.

The laser spot responds to the objects stored in the *Scene Module* and wraps around the object being pointed at. This feedback is intended to simplify the handling of the gesture. This concept is shown in Figure 14. If the pointing position is not changed and the gesture is not canceled, the object will be selected after a given time and highlighted in green by the projector. If the gesture is held further, the selection is sent to the *Interaction Module*.

The grid given by the virtual positions, from the *Scene Module*, facilitates selection and positioning. The circle adapts its shape to the grid when the user dwells on a position. The virtual object corresponding to the highlighted coordinate can now be used as either the source or destination. The selection done with the *spot* is saved in the scene.

### 5.7. Speech Module

**Figure 15:**
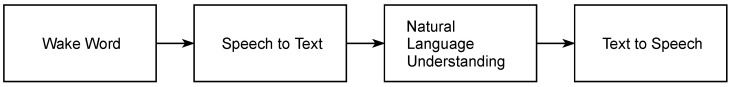
Common structure of speech assistants.

The *Speech Module*, along with the *Gesture Module*, is an important part of RoSA, that directly interacts with the user. As shown by Haeb-Umbach et al. [47], most established speech assistants consist of the modules shown in Figure 15. The individual components are explained in more detail below.

#### 5.7.1. Wake-Word Detection

Piccovoice was used to implement wake word detection. This application provides an online service for training personalized wake words. Furthermore, Piccovoice has a lower error rate compared to others [26]. The disadvantage of this implementation is that the software is only partially open-source and the use of the personalized wake words is limited to a 30-day license at a time.

#### 5.7.2. Voice Activity Detection (VAD)

Voice Activity Detection (VAD) is intended to prevent loud noises or the like from being interpreted as speech, e.g., after the system has been woken up by using the wake word [48]. In addition to activating STT, detection can be used as an abort criterion for the process. If a pause in speech exceeds a certain period of time, the sentence is terminated. We utilize a VAD developed by Google as part of the WebRTC project [28], which was intended to provide new standards for real-time communications with video, voice, and generic data support. It uses multiple frequency band features with a pre-trained Gaussian mixture model classifier.

#### 5.7.3. Speech-to-Text (STT)

For privacy reasons, an offline service based on Mozilla’s Deepspeech [49] is used. This open-source STT engine uses methods from Baidu’s research [27]. We used Deepspeech German, a pretrained network by Agarwal et al. [50]. The training data is based on Common Voice, a project started by Mozilla to collect speech data. It is also an open-source project that people donate their voice to, reading out sentences or validating audio transcripts. Since incorrect recognition of the STT can lead to difficulties in further processing, the vocabulary is adapted to that of the Natural Language Understanding (NLU) module.

To improve user experience and system accuracy, we introduced a visual feedback that displays the recognized spoken words to the user in real time. As suggested by Schurick et al. [51] this approach can greatly reduce the necessary time for speech input by a factor of three.

#### 5.7.4. Natural Language Understanding (NLU)

The system uses the open-source RASA solution to extract intent from text provided by the STT. RASA [29] consists of loosely coupled modules that combine a set of natural language processing and machine learning libraries into a unified API. It strives to balance adaptability and usability [52]. Braun et al. [53] show that the performance of Rasa NLU is compelling compared to several closed-source solutions. The NLU pipeline used consists of the SpacyNLP with the German language corpus, Tokenizer Featurizer, and EntityExtractor. First, the text is segmented by the Tokenizer, the Featurizer generates features for entity extraction and intention classification, and the EntityExtractor extracts information objects. The DIETClassifier is used to classify the intention. The classified intention and extracted information objects are sent to the *Interaction Module* as *Cube Action*.

Interaction with the robot works using *Cube Actions* (see Section 5.3.3). These can be categorized into the already mentioned, four basic and three complex operations. For each of these commands, there is a voice command. For example, the command “give me the white block with the letter A” combines the operations *pick* and *give*. The *Cube Action* passed through ROS looks like this:
Src.{letter"A",color"white"position[]}Dst.{letter[],color[],position[0,0,99]}.

In addition to voice-only commands, there is also the option of combining voice and gesture. The user uses the pointing gesture, selects a position and simultaneously specifies the desired block. “Place the black cube A here”. The *CubeAction* would look like this:
Src.{letter"A",color"black",position[]}Dst.{letter"spot",color[],position[]}.

Since the *Speech Module* has no information about user’s gesture input, the *Interaction Module* has to fill in the gaps and update the information using the *Gesture Module* and *Scene Module*.

By combining speech and gestures, ambiguous cases such as this can arise: “Give me the block”, which can mean handing over a block that has already been grabbed, but also *pick up* and *hand over* the block that is currently pointed to. The *CubeAction* passed via ROS nonetheless looks like this:
Src.{letter"spot",color[],position[]}Dst.{letter[],color[],position[0,0,99]}.

At this point, the *Interaction Module* must decide according to the *Scene Module* and *Cube Module* which action to perform.

From the four basic and three more complex operations, explained in Section 5.3.3, there is a set of 14 possible operations for manipulating the cubes using speech or speech and gesture combined. Since as many variations of the speech commands as possible are to be covered (e.g., instead of “cube”, “block”, or “square block”), the file generated from example sentences for training the NLU comprises 60,000 lines.

#### 5.7.5. Text-to-Speech (TTS)

The Windows Speech Application Programming Interface [54] is used for text to speech synthesis. This interface allows the user to make speaker variations such as:

- Audio Pitch: Determines the pitch (relative height or depth) of the speech synthesis.
- Include sentence boundary metadata: Determines whether sentence boundary metadata is added to a SpeechSynthesisStream object.
- Punctuation silence: Length of silence added after punctuation in SpeechSynthesis before another utterance begins.
- Speech rate: Sets the tempo, including pauses of the speech synthesis.

An advantage of this TTS system is that it is already integrated into Microsoft operating systems and is freely accessible since Windows Vista.

### 5.8. Interaction Module

**Figure 16:**
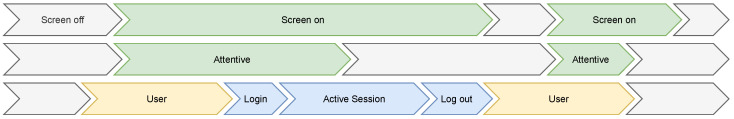
*Active Session* flow diagram.

The *Interaction Module* is adapted to the framework conditions of the individual workstations, since they require different user inputs and are to act independently of each other. At the beginning, the readiness for interaction is evaluated with features from the *Attention Module* and Kinect-stream. If a person is attentive, the corresponding workstation is activated and visual feedback is shown. Users identified by face recognition are signed in. The user-interface changes accordingly to the currently active user, settings, and last state of the workstation are restored. Users are signed in as long as they are attentive and recognized by the system. Identities assigned by the *Face Module* are applied to the Kinect skeleton with the largest intersection over union using the bounding box provided. Now, the user has a workstation bound *Active Session* until the user logs out (see Figure 16). This process is active on all workstations.

#### 5.8.1. WS1: Cubes and Cobot

If the WS is activated by the *Attention Module*, the user is informed via the screen that the authentication process is running, as long as no ID is available. If the user is still an unknown person, they will be prompted to register at WS2. Registered users are greeted with their stored name and a nod (quick up and down motions of the gripper) of the robot. At the first log-in, RoSA introduces itself to the user and runs through a basic tutorial. Once an *Active Session* is started at the workstation, the system can be interacted with. To issue a voice command, the wake word must be used. This activates the STT and the screen displays an icon of an earpiece to show the user that the system is now listening. The transcribed text is shown on the screen. Another icon indicates whether the use of a pointing gesture is possible. The gesture input is paused when the robot is already in motion so as to avoid problems with occlusion by the robot. Input via the pointing gesture results from holding the gesture in one position for a short time. The currently selected cube is also displayed on the screen.

#### 5.8.2. WS2: Registration

The interface of WS2 is based on ROS-QT (RQT), a development environment for visualization. The user is provided with an interface for registration. The data collected includes a name, preferred hand, and face recording. To record the face, the subject is asked to look first frontally and then once to the right and once to the left. The recorded data is stored in the database. Once this process is complete, an *Active Session* is started and the first part of the survey can begin. Upon successful completion of the first survey, a brief tutorial on RoSA and the tasks are presented. The user is prompted to perform the tasks on WS1. Finally, the second part of the questionnaire can be completed. When using WS2, the current progress of each user’s survey and tasks is saved. When logging in again, the display jumps to the last session. The user input comes from touch screen or a keyboard.

## 6. Experimental Studies

In order to prove the concept and to gather insight about necessary improvements for the system, a pilot study was conducted in the same manner as the previous WoZ study. During the study, data were collected from 11 subjects (2 ♀| 9 ♂) aged between 20 and 34 years. Five of these subjects had already participated in the previous RoSA study. The procedure of the study is as follows:

- Informed consent;
- WS2: Registration and collection of sociodemographic data;
- WS1: Collaborative tasks with robot;
  1. Have RoSA give you a block.
  2. Spell a specific word with alternating color of blocks.
  3. Build a 3-2-1-Pyramid with black-white-black layers.
- WS2: Questionnaires;
- WS1: Benchmark: Data collection for module assessment.

## 7. Results

Within the scope of this work, a functional robot system assistant is created. The system includes eight modules that communicate with each other. The system is activated by the *Attention Module* when a user shows enough willingness to interact. The *Face Module* allows a personalized user experience. The user is addressed personally and is shown personalized content. Both the voice and *Gesture Modules* can be used for intuitive operation of the robot. With the *Cube Module* and *Scene Module*, the system can interact with its environment. In addition, RoSA provides the user with auditory and visual feedback. The individual functions are explained to the user in a short tutorial, in the form of a self-introduction by RoSA, to start a natural dialogue.

### 7.1. Time

Table 2 summarizes the time needed for the completion of the collaborative tasks. Time was started as soon as the task was known and the user gave the first command, and stopped as soon as the task was declared complete by the experimenter.

All subjects were able to solve the tasks in collaboration with the robot in under two hours.

### 7.2. Questionnaires

The questionnaires (SUS [55], UMUX [56], PSSUQ [57], and ASQ [58]) were completed after the experiment. A module-specific questionnaire was then taken to additionally evaluate each module.

The summary of the user satisfaction questionnaires is presented in Table 3. The questionnaires were evaluated using the methods described in the literature for each individual. This mostly consisted of an alternating weighting of the questions, from which the mean value was calculated. To make the questionnaires comparable with each other, the scores were normalized by bringing them to the same scoring range of [0–100].

### 7.3. Modules

**Figure 17:**
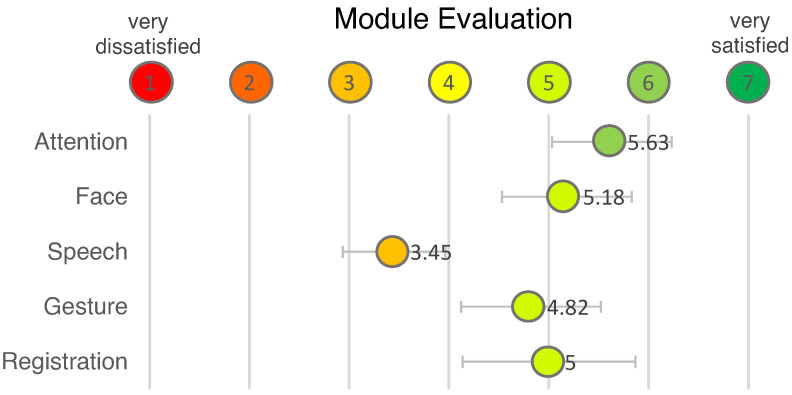
User rating of individual modules.

Users were also asked to rate each module according to their personal satisfaction on a scale of one, very dissatisfied, to seven, very satisfied (see Figure 17).

The test subjects were less satisfied with the *Gesture Module* and especially with the *Speech Module*. This is why we decided to evaluate these modules were tested independently of each other and outside the experiment to avoid external sources of error. The evaluation is based on the data that was collected during the benchmark phase at the end of the initial experiment with the cubes. The benchmark was run after the user had completed the questionnaires and already rated the system in so as to not bias the test subject.

### 7.4. Speech Module Evaluation

To evaluate the *Speech Module* the test subjects were asked to read out sentences displayed. For example: “Give me the white block with an A”.

The performance of a speech recognition system is measured by the Word Error Rate (WER):

(1) $$WER=\frac{S+I+D}{N}$$

where S is the number of words incorrectly replaced, I represents the number of additional words inserted, D is the number of words deleted, and N is the number of words correctly transcribed [59]. During the interaction, it was often the case that RoSA did not understand or misunderstood the subject. The error rate of the wake word detection was 33%.

The STT worked well overall. However, in some cases, words could not be detected because they were not part of the previously defined vocabulary. Short words like “yes” or single letters were often not recognized. Within the benchmark, the STT had a WER of 28.6%. The NLU module had an intent error rate of 27.3% and an entity error rate of 47.7%

### 7.5. Gesture Module Evaluation

For the evaluation of the pointing gestures, users were asked to point at targets highlighted on the screen in front of them, without any additional pointing feedback. The participants were asked to hold the gesture for two seconds. A 13-dot calibration pattern, as commonly used for eye-trackers, was used.

To estimate the pointing position, an intersection of a line, formed by two joints and a plane 2.5 m in front of the participant, was used. For each target, the timespan of one second, or equivalently 30 frames were evaluated and the resulting intersection points calculated. The spread, or the overall deviation of the positions from the calculated mean for each target, can be used as an estimation of the pointing quality.

**Figure 18:**
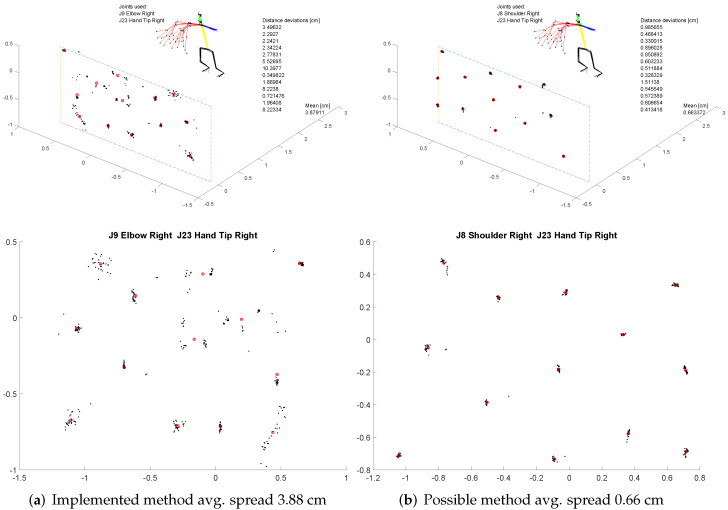
Evaluation of the spread of the resulting Points of Interest (POIs) at a 2.5-m distance. Calculated mean positions are depicted as red circle crosses.

As implied by the Laws of Linear HRI [12], any two joints can be used. The resulting intersection POIs that were calculated using joint pairs “Elbow Right—Hand Tip Right” as implemented in the experiment and “Shoulder Right—Hand Tip Right”, an alternative pointing method using the same skeletal data, can be seen in Figure 18.

The overall mean average of the position deviations is 3.88 cm for Elbow–Hand and 0.66 cm for Shoulder–Hand showing a possible way of decreasing the spread between the consecutively calculated pointing positions.

## 8. Discussion

In order to put the user data in perspective, they are compared with those of the WoZ study (see RoSA study [6]). It is important to note that the WoZ experiment was conducted under idealized conditions, demonstrating a close to flawless system adapting directly to the user’s preferred method of interaction.

Almost half of the participants in this study had already taken part in the WoZ study. Although the operating concepts could be individualized and freely chosen by the users and thus differed from the current scenario, an influence of the previous study cannot be ruled out.

Nonetheless, the participants were invited to aid in the discussion and evaluation of the system. Unfortunately, the sample size is still too small and the group of test subjects too homogeneous to be able to draw generally valid conclusions. However, qualitative statements can already be made about the system.

### 8.1. Efficiency

The system’s efficiency can be assessed by the time needed for the tasks. Table 4 shows the comparison to the WoZ study. It should be noted that during the WoZ study, the pyramid had to be built twice in the third task. However, across all tasks, the subjects in the WoZ study were faster.

The results in Table 2 show a large variance. The fastest user only needed one-sixth of the time for each task compared to the average user. Different times are required depending on the modality used. Since the pointing gesture can only express basic operations, hybrid or speech-based solution approaches are faster in theory. This is also true for the first task. The fastest person completed the task, using a voice command, within 16 s.

However, the more complex tasks showed that the error-proneness of the voice assistant caused severe delays. The person who completed the entire experiment the fastest used pointing gestures exclusively.

As explained earlier, errors occurred more frequently during the execution of the experiment. These negatively influenced the time needed for a task.

### 8.2. Usability

These aspects are also reflected in the user experience. The overall user satisfaction of the system turns out to be “satisfactory”. RoSA, in the current state, has visible deficiencies in the area of language that should be addressed.

Putting this study in context with the WoZ experiment, we find that the assessment is consistently unfavorable (see Table 5). This can be attributed to the lack of learning ability, higher susceptibility to errors, lower range of functions, and lack of personalization.

### 8.3. Modules

The connection of the individual components via middleware has weaknesses. Especially with large amounts of data such as video or audio data, high latency times can occur in the system. These lead to errors in modules that operate in a time-critical manner, such as attention monitoring.

The head pose recognition is insufficient to determine gaze direction and derive the user’s attention in certain scenarios. In addition to gaze direction, aspects such as proxemics and linguistic interaction should play a more important role. Drawing on more modalities increases the system’s robustness against failures such as loss of face detection.

Users were generally satisfied to very satisfied with attention detection. This module, in isolation, was one of the most robust. However, it is dependent on face recognition.

The face recognition faced problems with lighting conditions, covering of the face, and significant deviation of the angles. This would sometimes lead to loss of tracking and active session. The majority of subjects were satisfied with the registration process, while two subjects were rather dissatisfied. Both subjects had problems with the registration process because they were logged in as another user even before registration. The registration process was thus skipped and had to be initiated manually. The described classification error can be reduced by an improved initial recording of facial features; thus, different faces can be better differentiated.

As the first module in the speech processing pipeline, robust wake word detection is important. Experiments have shown that one third of the activations were not detected. Therefore, Wake-word activation needs to be improved by training on audio data. These problems might also be caused by the fact that for a system to accept the Wake-word, the user has to be logged in and attentive. In retrospect, this feature hinders the system’s intuitiveness, as the use of the Wake-word implies readiness for interaction. The inaccuracy of the STT is due to the reduction of the vocabulary by the known words. Likewise, the VAD sometimes causes very short words such as “yes” or single letters to be truncated. The benchmark results show that the NLU was partially able to compensate for STT errors by classifying the correct intent. However, due to the fact that the extraction of the entities had a high error rate and the created *Action* thus also contained errors, the commands could not be executed. The speech module is functional, but needs to be revised in its structure and handling to achieve good to very good results.

All users used the pointing gesture. This was necessary because stacking the pyramid was not possible using voice commands. However, this may also be due to the low error-proneness of the module. The support of the pointing gesture by the “laser pointer” was consistently seen as a relief. Users asked for the specification of two endpoints (start and end position) as a feature of the pointing gesture. Additionally, it was noted that when cubes were stacked on top of each other, the selection had to be made using the lowest position. One user described the laser pointer feedback as “shaky”. This refers to the pointer jumping back and forth between two grid positions. This user mainly used the wrist and index finger line for pointing. However, the implemented pointing gesture uses the elbow and wrist. Thus, the resulting line is inaccurate and leads to a more difficult selection. This is also shown by the data plots of the pointing gesture, showing that the pointing estimation using the shoulder leads to far better results. It could be possible to use an alternative algorithm using multiple joins (head, shoulder, elbow, hand, fingertip) instead of only two, to further increase the pointing estimation.

For more intuitive interaction, the dialog guidance should be further deepened. Currently, the system cannot fill in missing information on its own. For example, the dialog terminates if a command is not understood or only partially understood. Feedback is given to the user: “I didn’t understand that”. One solution would be for the system to output the specific error message. More intuitively, the missing information could be rephrased into a question.

## 9. Conclusions and Future Work

The presented implementation of the RoSA system is the first step from a simulated concept towards a real and functioning system.

The developed system meets the requirements for intuitiveness, which is confirmed by the study conducted. However, due to the limited number of participants, this study is only suitable as a pilot study to find errors in the system and optimize it. Furthermore, it shows how and what kind of data and streams could be gathered in future studies to further improve the system. The data from the benchmark was used to evaluate the system in its current state, but could be used to develop and test new methods. For example, the pointing gesture data could be used for a complex algorithm using multiple joints or for machine learning. Furthermore, the attention module could be enhanced by adding new features like body posture and distance.

The next upcoming studies and the updated RoSA system will include mobile robotics as additional non-stationary workstations to allow a higher adaptability of the system to real life scenarios and to improve the overall natural communication. For this, the system will be extended by the mobile robots Tiago (WS3) and Ari (WS4). These were developed by PAL-Robotics and can be seamlessly integrated through the ROS middleware.

For the future development of natural and collaborative human-robot interaction, a system is needed that can be further developed in a modular fashion and iteratively improved through ongoing studies and regular evaluations.

With RoSA, such a system has been created.

## Figures and Tables

**Table 1:** Extracted feature stream.

Stream	Feature	Description	Methods
Face	Face embedding Facial expression Face box Face center Facial landmark No. detected faces Face Id	512 features ∈[0,1] 7 features ∈[0,1] 4 features for each ∈(x,y) (in pixels) 1 features for each ∈(x,y) (in pixels) 5 features for each ∈(x,y) (in pixels) 1 feature $\in Z_{>0}$ 1 feature $\in Z_{>0}$	ArcFace [23] Residual Masking Network [24] RetinaFace [25] post processed RetinaFace [25] post processed post processed (cosine similarity)
Head	Head angles	3 features [yaw, pitch, roll] (in degrees)	Im2pose [25]
Gaze	Gaze direction Attention visual	2 features [yaw, pitch] (in degrees) 1 feature ∈0,1	Gaze360 [25] post processed
Speech	Wakeword Voice Activity Detection Speech-to-text Natural Language Processing	1 feature ∈0,1 1 feature ∈0,1 n features ∈“spoken text” 2 features ∈ [intent, entity]	Piccovoice [26] Deepspeech [27] WebRCT [28] RASA [29]
Distance	3D head position Face distance	3 features [x, y, z] 1 feature (in meter)	post processing using kinect post processed
Gesture	Hand Pose	4 Features (Open, Closed, Finger, None)	Kinect for Windows SDK 2.0
Body	Body Joints	26 Features [x, y, z]	Kinect for Windows SDK 2.0
Object	Cube Location	4 Features (Letter, Color, Bounding Box, Angle)	CubeDetector [30]

**Table 2:** Time needed to accomplish the tasks.

Variables	Fastest	Slowest	Mean
Task 1	00:00:16	00:04:30	00:01:21
Task 2	00:02:51	00:26:36	00:12:56
Task 3	00:02:36	00:36:39	00:11:06
Total	00:06:07	01:07:24	00:25:20

**Table 3:** Results of usability questionnaires.

Variablen	SUS [55]	UMUX [56]	PSSUQ [57]	ASQ [58]
Answer Range	1–5	1–7	1–7, NA	1–7, NA
Score Range	0–100	0–100	1–7	1–7
No. of Questions	10	4	16	3
Normalized Score	72.27	57.57	62.90	64.06
Total Avg. Score: 64.2				

**Table 4:** Comparison efficiency.

Variables	Task 1	Task 2	Task 3	Total
Results	00:01:21	00:12:56	00:11:06	00:25:20
WoZ study [6]	00:01:46	00:07:57	00:09:52	00:19:35
Deviation	00:00:25	−00:04:59	−00:01:08	−00:05:45

**Table 5:** Comparison usability.

Variables	SUS [55]	UMUX [56]	PSSUQ [57]	ASQ [58]
Results	72.27	57.57	62.90	64.06
WoZ study [6]	79.24	71.53	73.70	71.60
Deviation	6.97	13.96	10.8	7.54
Avg. deviation: 9.82				
